# Catalytic Wittig and aza-Wittig reactions

**DOI:** 10.3762/bjoc.12.253

**Published:** 2016-11-30

**Authors:** Zhiqi Lao, Patrick H Toy

**Affiliations:** 1 Department of Chemistry, The University of Hong Kong, Pokfulam Road, Hong Kong, People’s Republic of China

**Keywords:** aza-Wittig reactions, catalysis, phosphines, phosphine oxides, reduction, silanes, Wittig reactions

## Abstract

This review surveys the literature regarding the development of catalytic versions of the Wittig and aza-Wittig reactions. The first section summarizes how arsenic and tellurium-based catalytic Wittig-type reaction systems were developed first due to the relatively easy reduction of the oxides involved. This is followed by a presentation of the current state of the art regarding phosphine-catalyzed Wittig reactions. The second section covers the field of related catalytic aza-Wittig reactions that are catalyzed by both phosphine oxides and phosphines.

## Introduction

**Scheme 1:**
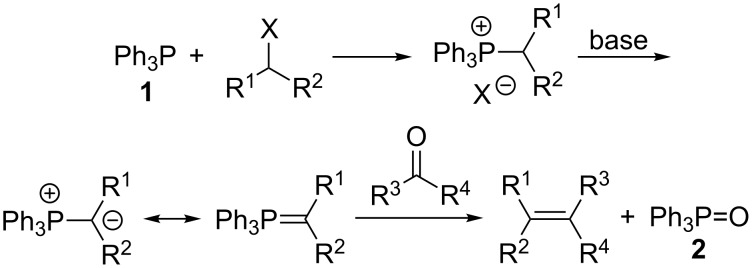
Prototypical Wittig reaction involving in situ phosphonium salt and phosphonium ylide formation.

The Wittig reaction is a venerable transformation for converting the carbon–oxygen double bond of an aldehyde or a ketone into a carbon–carbon double bond of an alkene group (Scheme 1). Since its introduction over half a century ago [1–2], it has been widely employed in organic synthesis due to its versatility and reliability. The requirement of simple and inexpensive reagents to generate the necessary phosphonium ylide (phosphorane) reactant (a phosphine, typically Ph_3_P (**1**), an alkyl halide and a base), also adds to its appeal [3–4]. However, despite its proven utility, the Wittig reaction suffers from limitations that may deter from its use, especially on a large scale, in the context of green sustainable chemistry. For example, it has low atom economy due to its requirement of one molar equivalent of a phosphine reagent, and the formation of a corresponding amount of a phosphine oxide, usually Ph_3_P=O (**2**). There is also the associated problem of separating a by-product from the desired product when they are formed in equal molar amounts. These major deficiencies of the Wittig reaction have led to numerous efforts towards developing variations of it which are catalytic in the required phosphine, or a surrogate for it, and this research is the major focus of this review [5–8]. Additionally, analogous catalytic aza-Wittig reactions, in which carbon–nitrogen double bonds of imine groups are formed, will also be discussed in the second section of this review.

## Review

### Catalytic Wittig reactions

A key requirement for versions of the Wittig reaction that are catalytic in phosphine is the selective in situ reduction of the P(V) phosphine oxide byproduct back to the P(III) phosphine in the presence of a reducible aldehyde or ketone substrate, an alkyl halide and a base. Thus, it seems that the challenge in developing catalytic versions of the Wittig reaction distils down to identifying and implementing selective reducing conditions that enables the necessary catalyst redox cycling, yet does not reduce either the starting materials or the desired alkene-containing product.

### Arsine and telluride-catalyzed reactions

**Scheme 2:**
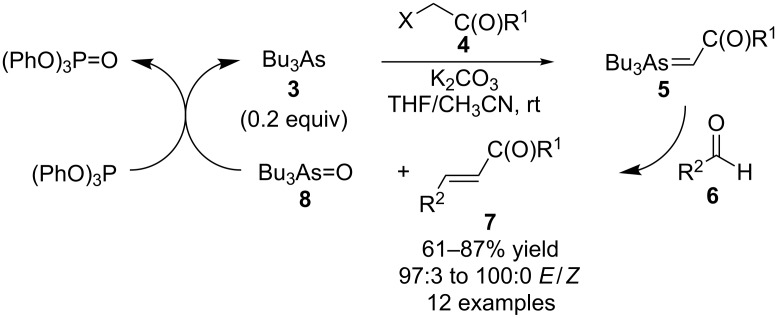
Bu_3_As-catalyzed Wittig-type reactions.

As phosphine oxides are generally very stable and relatively difficult to reduce, the group of Yao-Zeng Huang used their prior findings that arsonium ylides can participate in Wittig-type reactions. Further they found that arsine oxides can be reduced using much milder reaction conditions compared to phosphine oxides. They developed the first reported catalytic Wittig-type reactions in which Bu_3_As (**3**, 0.2 equivalents) was used as the catalyst (Scheme 2) [9–10]. The reaction of **3** with an alkyl halide **4** followed by deprotonation using potassium carbonate generated the corresponding arsonium ylide (**5**) which, in turn, reacted with an aldehyde substrate **6** to produce the alkene-containing product **7** together with Bu_3_As=O (**8**). The byproduct **8** was then reduced in situ using triphenylphosphite to regenerate catalyst **3** for participation in another reaction cycle. Overall, the reaction conditions were quite mild, with the reactions being performed at room temperature with only slight excesses of base and reducing reagent being required. It should be noted that the use of only electron-withdrawing groups activated alkyl halides **4**, and that aromatic and aliphatic aldehydes **6** worked well in these reactions to produce products **7** in high yields with predominantly *E*-configuration.

**Scheme 3:**
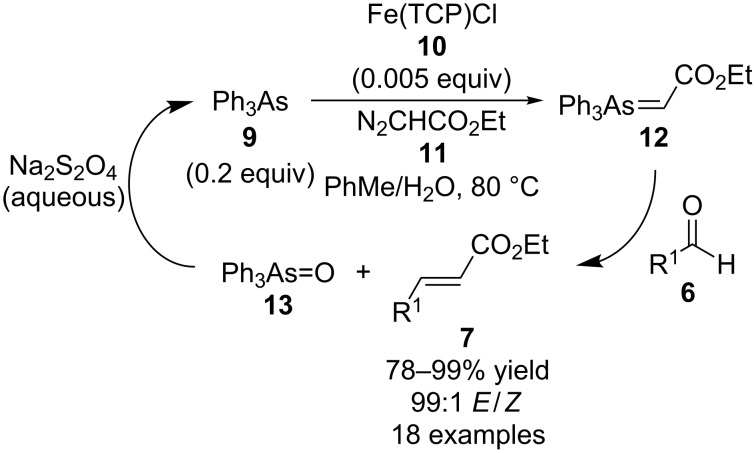
Ph_3_As-catalyzed Wittig-type reactions using Fe(TCP)Cl and ethyl diazoacetate for arsonium ylide generation.

Quite a few years later Yong Tang and co-workers, also of the Shanghai Institute of Organic Chemistry, carried on with this research and extended it by using a combination of Ph_3_As (**9**, 0.2 equivalents), Fe(TCP)Cl (**10**, TCP = tetra(*p*-chlorophenyl)porphyrinate), and ethyl diazoacetate (**11**) to generate arsonium ylide **12** for use in biphasic catalytic Wittig-type reactions (Scheme 3) [11]. In these reactions sodium hydrosulfite replaced triphenylphosphite as the reducing reagent to convert the byproduct Ph_3_As=O (**13**) back into **9** in the aqueous phase of the reaction mixture in order to make the reactions more environmentally friendly. As was the case in the previous work described above, both aromatic and aliphatic aldehydes **6** were suitable substrates in this reaction system to form products **7**.

**Figure 1:**
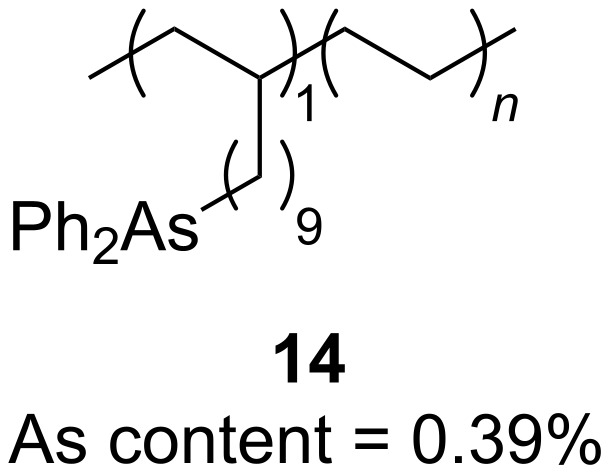
Recyclable polymer-supported arsine for catalytic Wittig-type reactions.

Most recently the Tang research group has reported the use of polymer-supported arsine **14** (0.008–0.04 equivalents) as the catalyst in related reactions (Figure 1) [12]. In this work, **14** was found to be the only arsine examined that was able to effectively catalyze Wittig-type reactions of ketone substrates to produce tri- and tetrasubstituted alkene products in very high yields. For these reactions, which required a higher operating temperature than before (110 °C compared to 80 °C), polymethylhydrosiloxane was used as the reductant, and **14** could be recovered and reused efficiently in numerous reaction cycles without loss of catalytic activity.

**Scheme 4:**
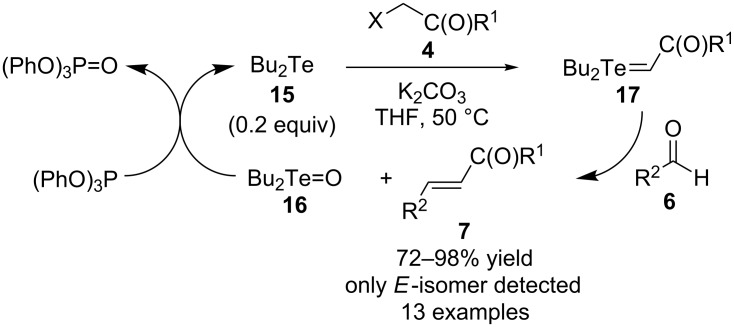
Bu_2_Te-catalyzed Wittig-type reactions.

At about the time that Huang and co-workers reported their catalytic reactions using **3** (Scheme 2), they also disclosed that Bu_2_Te (**15**) could function similarly as a catalyst in such Wittig-type reactions due to the relatively weak tellurium–oxygen bond of dialkyl telluroxides, such as Bu_2_Te=O (**16**) (Scheme 4) [13]. Aldehydes **6** were again used as substrates in reactions to form products **7**. No comments were made regarding the relative advantages or disadvantages of using either **3** or **15** as the catalyst for such reactions, and in fact similar alkyl halides **4** were used to generate ylides **16** as were used in the reactions catalyzed by **3**. The only notable differences regarding performing the reactions were that reactions with **15** required a higher temperature (50 °C compared to room temperature) and acetonitrile as a co-solvent. However, product yields and stereoselectivities were slightly improved when **15** was used at the same loading level (0.2 equivalents) and with a similar set of aldehyde **6** substrates.

**Scheme 5:**
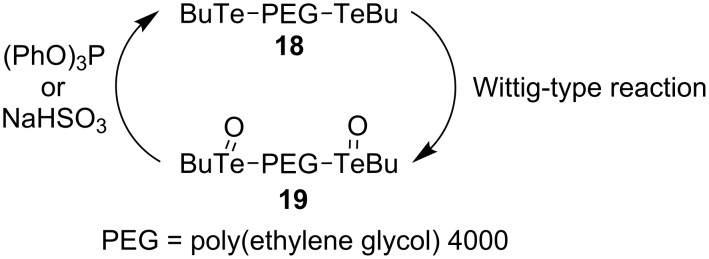
Polymer-supported telluride catalyst cycling.

Tang’s research group also followed up this tellurium-based research many years later and published several papers describing the use of polymer-supported tellurides, such as **18**, as catalysts (Scheme 5) [14–16]. The major advantage reported for using **18** instead of **15** is that a much lower catalyst loading could be used in similar reactions (0.02 equivalents compared to 0.2 equivalents). Unfortunately, despite the fact that **19** could be easily reduced to **18** using triphenylphosphite or sodium bisulfite, and simply removed from the desired alkene products, recovered **18** had a much lower activity when attempts to reuse it were made. Nevertheless, the use of **18** with sodium bisulfite as the reducing reagent allowed for very simple product isolation, and reaction yields and stereoselectivities were similar to when **15** was used as the catalyst.

**Scheme 6:**
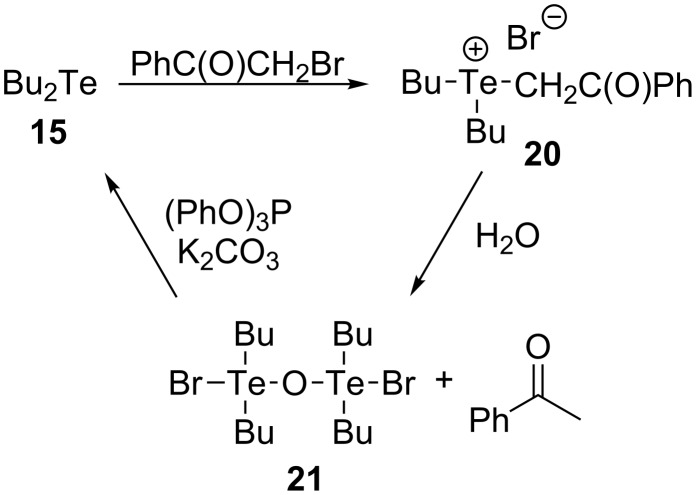
Stable and odourless telluronium salt pre-catalyst for Wittig-type reactions.

In the course of performing the research mentioned above, Tang and co-workers made the fortuitous observation that telluronium salt **20** (prepared from **15**) decomposed in the presence of water to form **21**. This compound could be used as a pre-catalyst in Wittig-type reactions because it is reduced to **15** by triphenylphosphite in the presence of potassium carbonate (Scheme 6) [17]. Since **21** was observed to be stable and odourless, its use has some practical advantages, and when it was used as a surrogate for **15**, very similar results were obtained.

While these arsenic and tellurium-based reactions are conceptually interesting and show the way in which phosphorous-based catalytic Wittig reactions might be performed, it appears that they have not been used by anyone other than the original reporters.

### Phosphine-catalyzed reactions

As alluded to above, it seems that the major impediment to the development of phosphine-catalyzed Wittig reactions was the stability of phosphine oxides and the harsh reaction conditions generally required to reduce them to the corresponding phosphines were thought to be incompatible with various necessary reactants and reagents, and the desired reaction products to be formed.

**Scheme 7:**
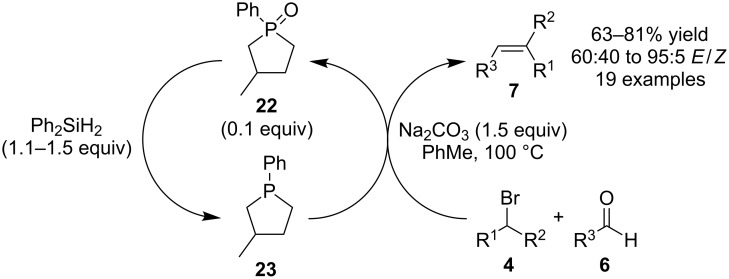
Phosphine-catalyzed Wittig reactions.

Christopher J. O’Brien and co-workers recently reported a breakthrough of identifying the necessary selective conditions for phosphine oxide reduction that allowed phosphorous-based catalytic Wittig reactions to become realized. In their initial publication they described the use of readily available phosphine oxide **22** as a pre-catalyst (0.1 equivalents), which was reduced in situ with diphenylsilane (Ph_2_SiH_2_) to phosphine **23**, which served as the actual catalyst in their Wittig reactions (Scheme 7) [18]. Once **23** was generated, it reacted with electron-withdrawing group activated alkyl halides **4** and sodium carbonate to form the corresponding phosphonium ylides that reacted with aldehydes **6** to produce alkene products **7** and **22** as a byproduct.

**Figure 2:**
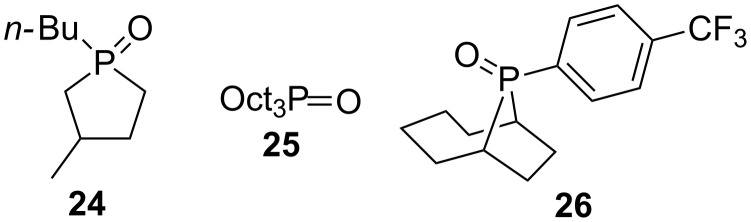
Various phosphine oxides used as pre-catalysts.

Subsequently they reported that the soluble organic base *N*,*N*-diisopropylethylamine was a good replacement for sodium carbonate in such reactions [19], and that the addition of 4-nitrobenzoic acid facilitated the phosphine oxide reduction step using phenylsilane (PhSiH_3_) instead of diphenylsilane [20]. Using this combination of 4-nitrobenzoic acid and phenylsilane for phosphine oxide reduction allowed reactions starting with phosphine oxide **24** (Figure 2) to be conducted at room temperature and for acyclic phosphine oxides **2** and **25** to be used as well, albeit at elevated operating temperature. Most recently they have found that the use of **26** as the pre-catalyst in conjunction with sodium *tert*-butoxycarbonate (NaOCO_2_*t-*Bu, a slow release form of sodium *tert*-butoxide) as a precursor for the required base, catalytic Wittig reactions could be performed using semi or non-stabilized ylides [21].

**Scheme 8:**
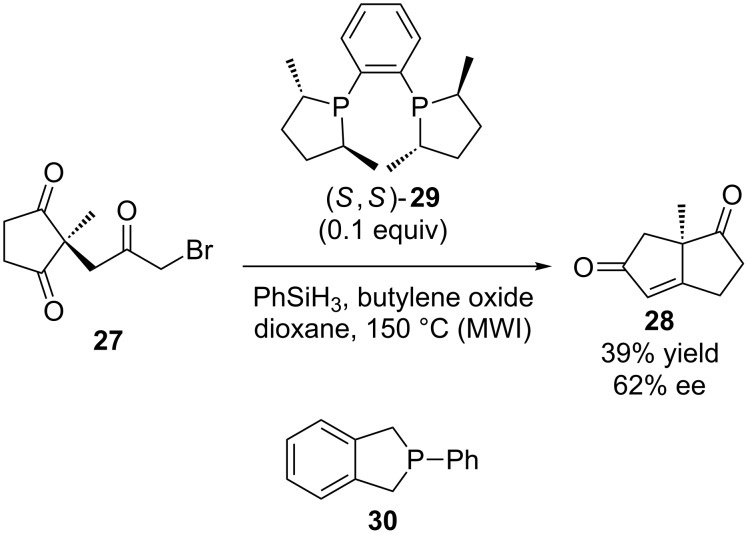
Enantioselective catalytic Wittig reaction reported by Werner.

Thomas Werner’s research group has also been active in this area of research, and reported the first example of a catalytic enantioselective Wittig reaction (Scheme 8) [22]. This reaction involved the intramolecular cyclization of **27** to form **28**. A variety of phosphines were examined as the catalyst, and (*S*,*S*)-**29** ((*S*,*S*)-Me-DuPhos, 0.1 equivalent) was found to provide the best combination of reactivity and stereoselectivity (39% yield, 62% ee). In these reactions butylene oxide was used as a base precursor, phenylsilane was the reducing reagent, and the reactions were performed using microwave irradiation (MWI). Subsequently, Werner et al. reported the scope and limitations of such microwave-assisted catalytic Wittig reactions using tributylphosphine, phenylsilane and butylene oxide [23–24], and reactions with the combination of achiral **30**, trimethoxysilane ((MeO)_3_SiH) and sodium carbonate using conventional heating [25].

**Scheme 9:**
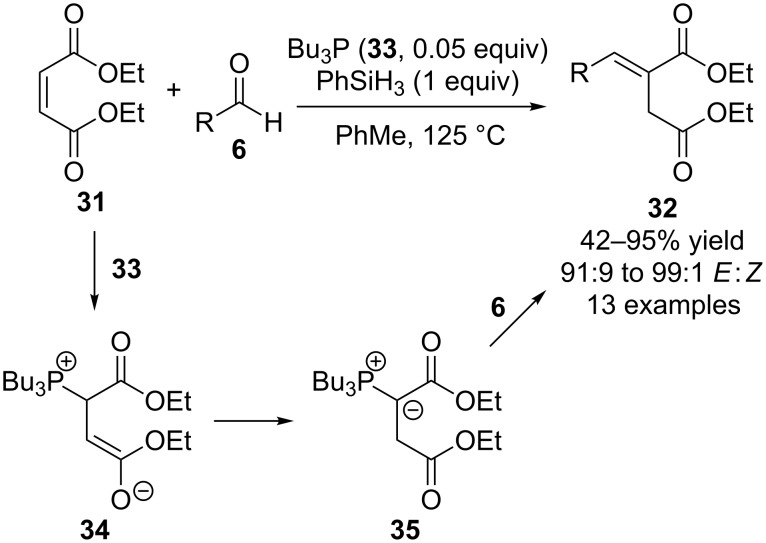
Base-free catalytic Wittig reactions reported by Werner.

Additionally, the same authors have reported base-free Wittig reactions using diethyl maleate (**31**) as the starting material to form products **32** catalyzed by tributylphosphine (**33**, 0.05 equivalents) (Scheme 9) [26]. In these transformations the initial reaction between **31** and **33** generated zwitterion **34**, that underwent internal proton transfer to generate ylide **35**. This in turn reacted with aldehyde **6** to form **32** and phenylsilane was used as the reducing reagent to regenerate **33**. Most recently they refined such reactions using **22** as the pre-catalyst, trimethoxysilane as the reducing reagent, and catalytic benzoic acid to facilitate phosphine oxide reduction [27].

**Scheme 10:**
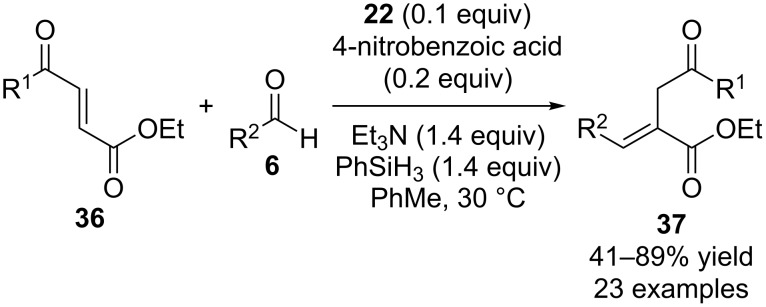
Catalytic Wittig reactions reported by Lin.

At about the same time as the penultimate report by Werner and co-workers appeared, Wenwei Lin and a co-worker published conceptually similar catalytic Wittig reactions that were based on their previous research regarding related non-catalytic phosphine-mediated base-free Wittig reactions (Scheme 10) [28]. They started with Michael acceptors **36** to generate products **37** using **22** as the pre-catalyst with triethylamine as the base, phenylsilane as the reducing reagent, and 4-nitrobenzoic acid as an acidic additive. It should be noted that the role of the base in these reactions is unclear and not directly commented on. According to the proposed mechanism for the formation of the required ylide intermediate, a base is not necessary, but the authors reported that when it was omitted from a control reaction, no reaction occurred.

**Scheme 11:**
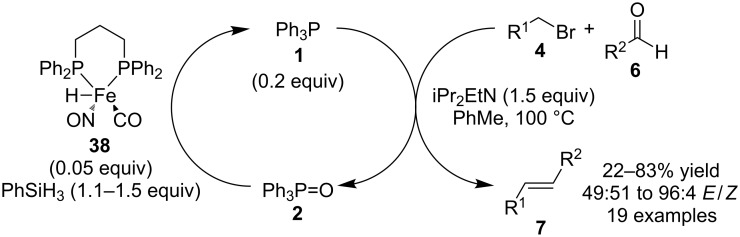
Catalytic Wittig reactions reported by Plietker.

Finally, Bernd Plietker and co-workers have very recently reported the use of iron complex **38** as a catalyst for phosphine oxide reduction and have incorporated it into Wittig reactions catalyzed by **1** (Scheme 11) [29]. These reactions are similar to those mentioned previously by O’Brien’s group [20] that involve the cycling between **1** and **2** using a silane reducing reagent.

As can be seen above, the issue of selective phosphine oxide reduction has been solved using various silane reagents and much progress has been made in phosphine-catalyzed Wittig reactions. Initial results were reported using phosphine oxides that were prepared from commercially available phosphine oxide starting materials that were relatively easy to reduce, such as **22**. However, relatively mild cycling between **1** and **2** can now be achieved, and this may make such catalytic Wittig reactions more popular, practical, and scalable due to the stability, wide availability and low cost of **1**.

### Catalytic aza-Wittig reactions

**Scheme 12:**
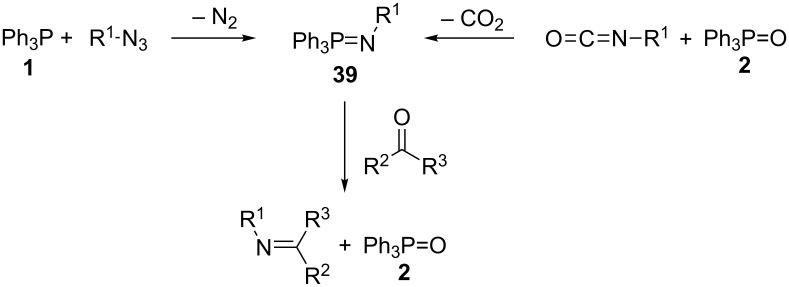
Prototypical aza-Wittig reaction involving in situ iminophosphorane formation.

Aza-Wittig reactions are similar to Wittig reactions in that they also involve the reaction of a phosphonium ylide, in this case an iminophosphorane (or phosphinimide) such as **39**, with a carbonyl group containing compound to form the carbon–nitrogen double bond of an imine along with a byproduct phosphine oxide such as **2** (Scheme 12). The difference is that the iminophosphorane reagents can be generated either from a phosphine such as **1** or from a phosphine oxide such as **2**, by reaction with either an azide or isocyanate reagent, respectively. Thus, two possible strategies for catalytic aza-Wittig reactions exist, one using a phosphorous(V) catalyst, and the other using a phosphorous(III) catalyst that is regenerated in the catalytic cycle. These strategies are the topic of this section of the review.

### Phosphine oxide-catalyzed reactions

**Scheme 13:**
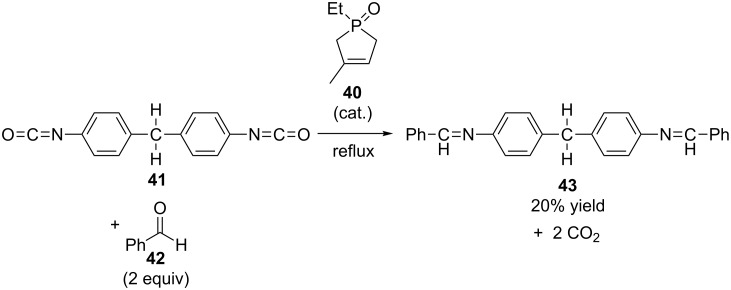
First catalytic aza-Wittig reaction reported by Campbell.

It is clear from Scheme 12 that when a phosphine oxide is used to generate the iminophosphorane **39** for an aza-Wittig reaction, it is regenerated as a byproduct, and thus can participate directly in another reaction cycle. More than 25 years before Huang’s research group made their first report regarding arsine catalysis [10], Tod W. Campbell and colleagues took advantage of this fact and reported a single example of a catalytic aza-Wittig reaction as part of their research on phosphine oxide-catalyzed carbodiimide synthesis (Scheme 13) [30]. In their reaction they used phosphine oxide **40** as the catalyst in the reaction between diisocyanate **41** and benzaldehyde (**42**, 2 equivalents) to form diimine **43** and carbon dioxide (2 equivalents). While this reaction was described as being catalytic, the mass of the catalyst used was not explicitly reported, so it is impossible to determine the number of catalyst turnovers that were involved in generating the 20% isolated yield of **43**. The authors only reported that “one drop” of **40** was used in a reaction starting with 7 grams of **41**.

**Scheme 14:**
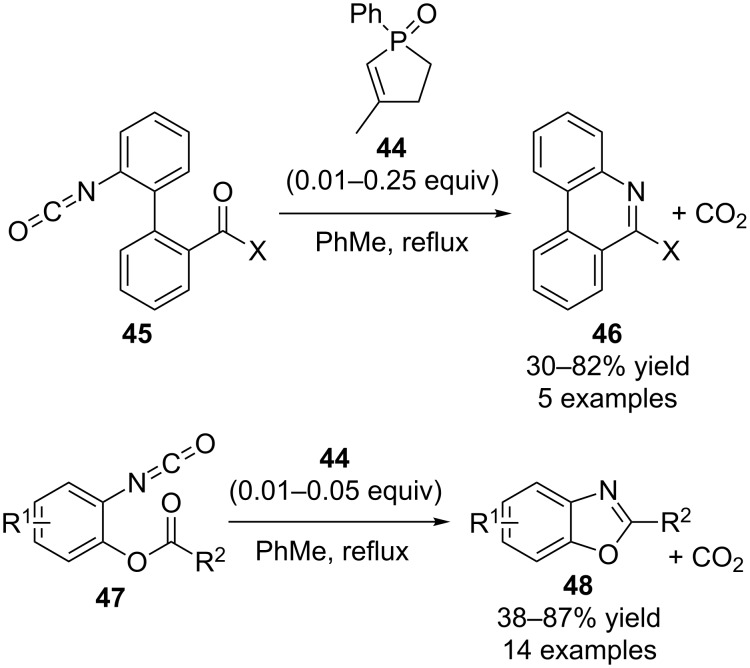
Intramolecular catalytic aza-Wittig reactions reported by Marsden.

Subsequently, Stephen P. Marsden and co-workers reported intramolecular versions of catalytic aza-Wittig reactions for heteroaromatic compound synthesis using commercially available phosphine oxide **44** as the catalyst (Scheme 14) [31]. In this work, biaryl isocyanates **45** could be converted into phenanthridines **46**, and aryl isocyanates **47** could be transformed into benzoxazoles **48** directly in refluxing toluene together with the simultaneous release of carbon dioxide. Presumably these reactions proceeded via iminophosphorane intermediates that reacted intramolecularly with the carbonyl groups to form the obtained cyclic products. It is noteworthy that the loading of catalyst **44** could be as low as 0.01 equivalent.

**Scheme 15:**
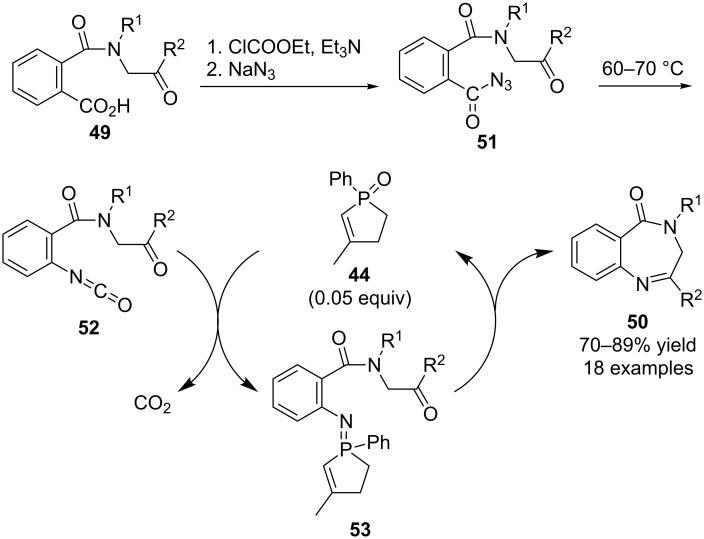
Catalytic aza-Wittig reactions in 1,4-benzodiazepin-5-one synthesis.

More recently the research group of Ming-Wu Ding has extended this concept of phosphine oxide-catalyzed aza-Wittig reactions to the conversion of carboxylic acid derivatives **49** into 1,4-benzodiazepin-5-ones **50** using catalyst **44** (Scheme 15) [32]. The overall transformations were reported to occur via acyl azide intermediates **51** that were not purified, but instead used directly in thermal Curtius rearrangement reactions that afforded isocyanates **52**. These were in turn treated in situ with catalyst **44** to afford the final products **50** via presumed iminophosphorane intermediates **53**.

**Scheme 16:**
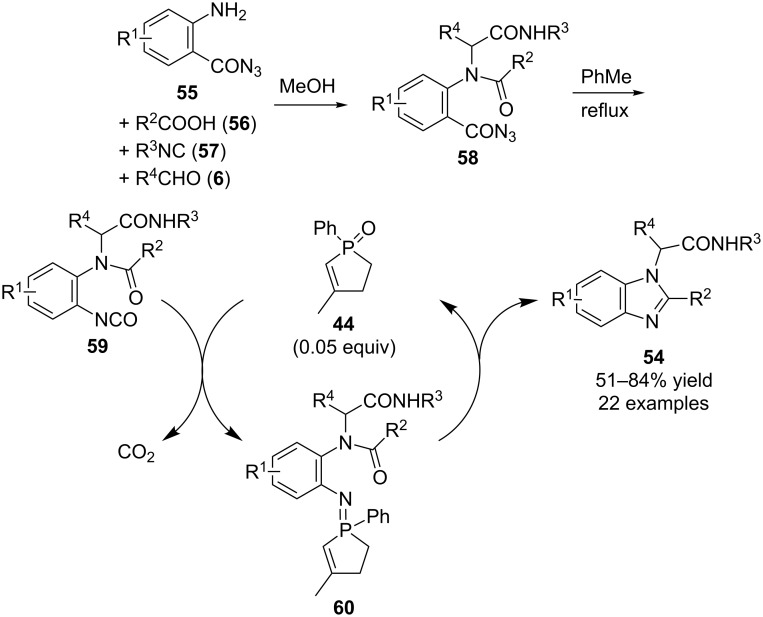
Catalytic aza-Wittig reactions in benzimidazole synthesis.

Subsequently this research group used a very similar strategy for the synthesis of polysubstituted benzimidazoles **54** via sequential Ugi and catalytic aza-Wittig reactions (Scheme 16) [33]. It was reported that mixing 2-aminobenzoyl azides **55**, carboxylic acids **56**, isocyanides **57** with aldehydes **6** in methanol generated intermediates **58**, which underwent rearrangement to isocyanates **59** in refluxing toluene. Finally, catalytic aza-Wittig reactions with **44** produced cyclized final products **54** via iminophosphoranes **60**.

### Phosphine-catalyzed reactions

**Scheme 17:**
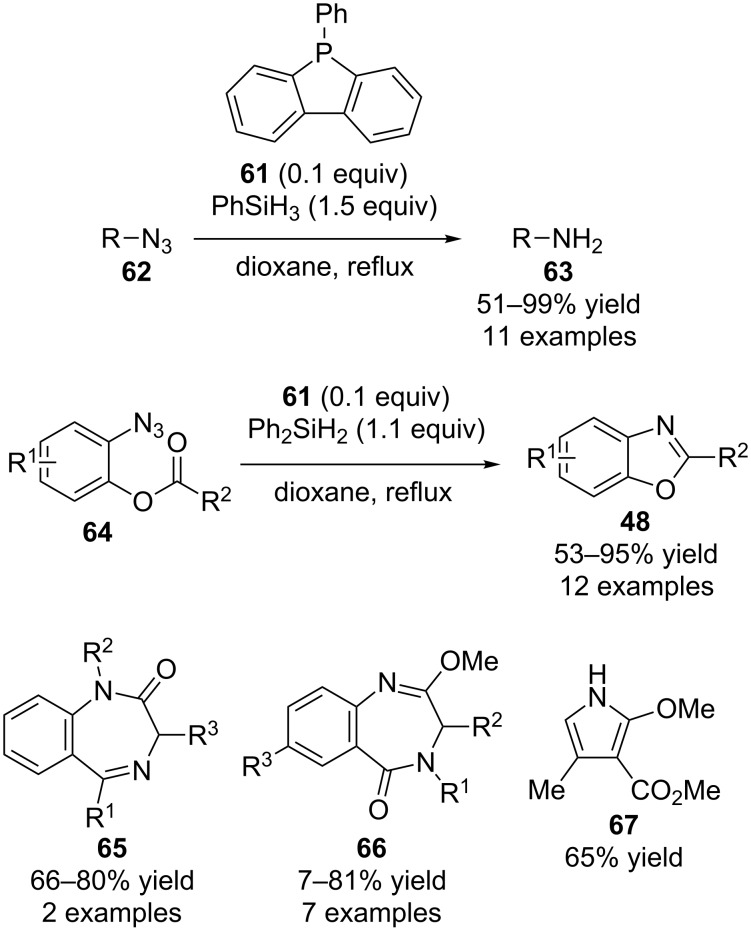
Phosphine-catalyzed Staudinger and aza-Wittig reactions.

The initial research in this area was performed by Floris L. van Delft and co-workers who reported the synthesis of **61** and its use in catalytic Staudinger reactions for the reduction of azides **62** to amines **63** (Scheme 17) [34]. Subsequently they extended such reactions to include aza-Wittig reactions using diphenylsilane as the reducing reagent [35]. For example, starting materials **64** could be converted into benzoxazoles **48** in overall net transformations that were similar as to those discussed above by Marsden (Scheme 14). In their report they also described the synthesis of various other classes of heterocyclic compounds such as 3*H*-1,4-benzodiazepin-2(1*H*)-ones **65**, 3*H*-1,4-benzodiazepin-5(4*H*)-ones **66**, and pyrrole **67** from the corresponding starting materials using similar reaction conditions.

**Scheme 18:**
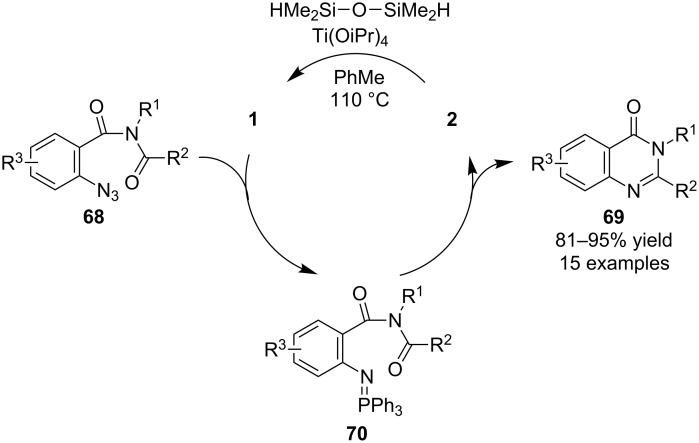
Catalytic aza-Wittig reactions in 4(3*H*)-quinazolinone synthesis.

In addition to phosphine oxide-catalyzed aza-Wittig reactions, Ding’s research group has also explored the use of phosphine catalysis in such reactions. In their initial report regarding this strategy, they used **1** to catalyze intramolecular reactions that converted aryl azides **68** into 4(3*H*)-quinazolinones **69** via intermediate iminophosphoranes **70**, using the combination of titanium tetraisopropoxide and tetramethyldisiloxane (TMDS) for the in situ reduction of byproduct **2** (Scheme 18) [36].

**Scheme 19:**
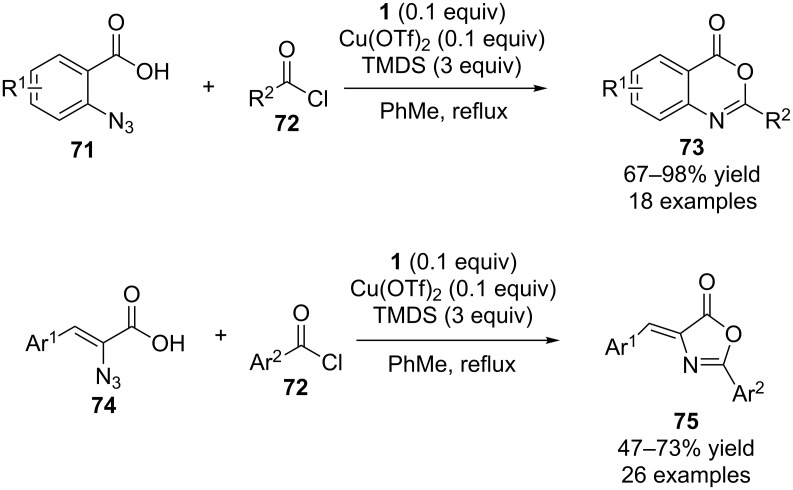
Catalytic aza-Wittig reactions of in situ generated carboxylic acid anhydrides.

This group has more recently studied catalytic aza-Wittig reactions using carboxylic acid anhydrides as the starting materials (Scheme 19) [37]. For example, reactions of carboxylic acids **71** with acid chlorides **72** to generate the corresponding carboxylic acid anhydride in situ afforded 4*H*-benzo[*d*][1,3]-oxazin-4-ones **73**. In these reactions, **1** was used as the catalyst for the aza-Wittig reaction and copper triflate was used as the catalyst for phosphine oxide reduction. Using similar conditions the corresponding reactions of carboxylic acids **74** with aromatic acid chlorides **72** produced 4-benzylidene-2-aryloxazol-5(4*H*)-ones **75**.

**Scheme 20:**
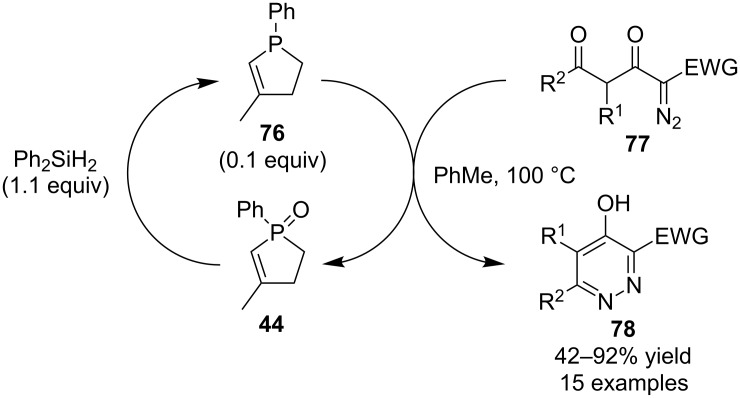
Phosphine-catalyzed diaza-Wittig reactions.

Lastly, the research group of Piet Herdewijn extended this general concept and reported catalytic diaza-Wittig reactions (Scheme 20) [38]. In these reactions **76** (from the reduction of **44**) was the catalyst that transformed diazo group containing starting materials **77** into pyridazine derivatives **78**. In these reactions **44** was actually added to the reaction mixtures as the pre-catalyst that was reduced using diphenylsilane.

It is readily evident from the above examples that regardless of whether phosphine or phosphine oxides were used as the catalyst, catalytic aza-Wittig reactions have emerged to become powerful tools in the synthesis of collections of various heterocyclic compounds since it seems that the required isocyanate or azide group containing precursors are readily synthesized from simple starting materials.

## Conclusion

While great advances have been reported regarding the development of catalytic Wittig and aza-Wittig reactions, it remains to be seen how widely these methods will be adopted. Evidence that the former reactions are being described in the literature with increasing frequency are the very recent reports by Saleh and Voituriez [39], and Wenwei Lin and co-workers [40] regarding intramolecular reactions to form heterocycles that appeared as this review was being completed. However, will the research results summarized herein remain merely intellectual achievements or will they become commonly used synthetic methods in the future? Perhaps the answer to this question is how these new reaction systems are viewed from an environmental/green chemistry perspective. In this regard it is encouraging that a life cycle assessment indicates that the use of stoichiometric quantities of silanes as replacements for phosphines in catalytic Wittig reactions can offer environmental improvements [41]. Thus it seems somewhat reasonable to expect that as even more efficient methods for phosphine oxide reduction are discovered [42–47], catalytic reactions involving cycling between phosphines and phosphine oxides will become more environmentally friendly and more popular too. With regards to the phosphine oxide-catalyzed aza-Wittig reactions discussed, while no life cycle assessments have been performed, they do seem to be rather “green” since no redox cycling is necessary, and there appear to be few constraints to their potential application.
